# Induction of Different Sensitization Patterns of MRSA to Antibiotics Using Electroporation

**DOI:** 10.3390/molecules23071799

**Published:** 2018-07-20

**Authors:** Vitalij Novickij, Jurgita Švedienė, Algimantas Paškevičius, Svetlana Markovskaja, Eglė Lastauskienė, Auksė Zinkevičienė, Irutė Girkontaitė, Jurij Novickij

**Affiliations:** 1 Institute of High Magnetic Fields, Vilnius Gediminas Technical University, Naugarduko st. 41, 03227 Vilnius, Lithuania; jurij.novickij@vgtu.lt; 2 Laboratory of Biodeterioration Research, Nature Research Centre, Akademijos st. 2, 08412 Vilnius, Lithuania; jurgita.svediene@gamtc.lt (J.Š.); algimantas.paskevicius@gamtc.lt (A.P.); 3 Laboratory of Microbiology of the Centre of Laboratory Medicine, Vilnius University Hospital Santariškių Clinics, Santariskiu g. 2, 08406 Vilnius, Lithuania; 4 Laboratory of Mycology, Nature Research Centre, Žaliųjų ežerų st. 49, 08406 Vilnius, Lithuania; svetlana.markovskaja@gamtc.lt; 5 Department of Microbiology and Biotechnology, Vilnius University, Sauletekio al. 7, 10257 Vilnius, Lithuania; egle.lastauskiene@gf.vu.lt; 6 State Research Institute Centre for Innovative Medicine, Department of Immunology, Santariškių st. 5, 08406 Vilnius, Lithuania; aukse.zinkeviciene@imcentras.lt (A.Z.); irute.girkontaite@imcentras.lt (I.G.)

**Keywords:** antibiotics, electric field, drug resistance, membrane permeabilization, *S. aureus*

## Abstract

Treatment of bacteria-associated infections is complicated and antibiotic treatment alone is often inadequate to overcome biofilm infections. Physical methods allow overcoming this problem and propose solutions that are non-dependent on drug resistance. In this work, we investigated the feasibility of pulsed electric fields for sensitization of MRSA to common antibiotics. We analyzed the efficacy of inactivation of methicillin-resistant *Staphylococcus aureus* in 5–20 kV/cm electric field separately and in combination with gentamicin, doxycycline, ciprofloxacin, sulfamethoxazole, and vancomycin. Combined treatment allowed using up to 1000-fold smaller concentrations of antibiotics to induce the same inactivation of *S. aureus*.

## 1. Introduction

The development of drug resistance in bacteria is a worldwide problem, while *Staphylococcus aureus* (*S. aureus*) is remarkable in its ability to acquire resistance to any antibiotic [1]. As a result, infections, which are caused by methicillin-resistant strains of *S. aureus* (MRSA) have reached epidemic proportions globally and the World Health Organization (WHO) has ranked it in the list of top five bacteria that pose the greatest threat to human health and for which new antibiotics and/or combined antimicrobial techniques are desperately needed [2,3].

Currently, antibiotics are routinely used in hospitals and intensive care units for biocontrol of microbial infections [4,5]. Nevertheless, the resistance of MRSA to many antibiotics and the capability to form biofilms makes the treatment of *S. aureus-*associated infections challenging [6]. The failure of any chemical antimicrobial therapy is highly associated with bacteria biofilm formations, while persistent infections are characterized by increased morbidity and mortality [7]. In the case of systemic infections, antibiotic concentrations can be up to 1000-fold higher than the therapeutic concentrations, which are used for single-cells of the same species, thus development of new, drug-resistance independent methods is needed [8].

To address the problem, an increasing number of laboratories study the effects of physical methods for the effective inactivation of bacteria [9,10,11]. Ermolaeva et al. have shown that atmospheric cold plasma causes intracellular damage of *S. aureus* and allows inactivating bacteria by strong oxidative stress [12]. As an alternative, the potency of photodynamic therapy was highlighted by Almeida et al. and a first report on the efficacy of curcumin showed that photodynamic therapy may be effective against MRSA infections [13]. Reports on the bioelectric inhibitory effects of low and high frequency electric currents are also available [14,15]. Nevertheless, the available methodologies have limitations such as a long time of exposure [14], the requirement to use photosensitizers [13] or other undesired side effects [16]. In this study, we extend the array of effective bacteria inactivation methodologies by providing data on the inactivation efficacy of electroporation in combination with typical antibiotics.

Electroporation (EP) is a pulsed electric field (PEF) induced phenomenon of cell wall and membrane permeabilization [17], resulting in the increase of molecules transfer to which the membrane was initially impermeable. The phenomenon is also pulse dependent, which implies that the damage to the cell is caused by PEF scales with delivered pulse energy [18]. Depending on the treatment intensity the described membrane permeabilization can be transient and cells survive or it can lead to cell death and is then referred to as irreversible electroporation [19]. In both cases, the methodology has found many applications in biotechnology and biomedicine, predominantly in cancer treatment [20,21]. It should be noted that electroporation employs high intensity electric fields and the mechanism of effect is fundamentally different from the available reports on low intensity electric field (1000-fold lower) effects and low current methodologies [14,15,22]. The EP is rapid, non-thermal and the intensity can be precisely controlled, while its physical nature makes it impossible for the microorganisms to develop resistance. It is widely used for transformation of bacteria [23] or sterilization of liquid food [24]. However, the number of works focusing on the potential of EP for treatment of surface infections and multi-drug resistant bacterial strains is very limited. It is an emerging field and the feasibility of electroporation for treatment of surface bacterial infections, wound sterilization, and biofilm eradication was highlighted only recently and the first proof of concept was shown both in vitro and in vivo [25,26,27]. At the current state of knowledge, the additive effect of EP with antibiotics is unknown, however, in our previous work we have shown a proof of concept that electroporation can be used to sensitize drug-resistant *Candida albicans*, that is, PEF can be used to increase the sensitivity of pathogens to drugs to which the strains are initially resistant [28]. Since the biological factors that influence the susceptibility of the microorganisms to PEF inactivation and/or permeabilization are related only to the morphological and physiological parameters of the treated cells, we speculated that a similar drug sensitization phenomenon may be apparent in bacteria.

Therefore, in this work, we investigated the feasibility of PEF technology for sensitization of MRSA to common antibiotics. We analyzed the efficacy of inactivation of MRSA in 5–20 kV/cm electric field separately and in combination with gentamicin, doxycycline, ciprofloxacin, sulfametoxazole and vancomycin. It is the first study analyzing the PEF-induced antibiotics sensitization patterns and the results could be useful for development of new antimicrobial techniques for treatment of infections, which are caused by drug-resistant strains.

## 2. Results

**Figure 1:**
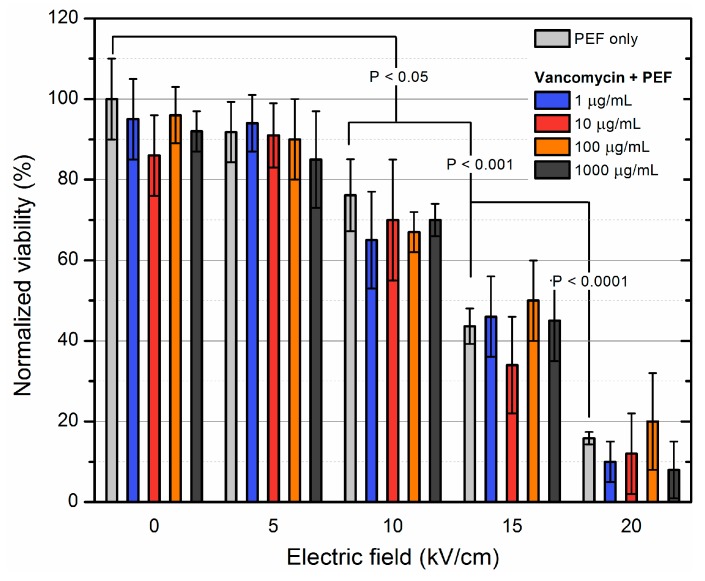
Inactivation of MRSA using electroporation separately and in combination with vancomycin of varied concentration. The 10 kV/cm PEF amplitude was the threshold value to induce inactivation of MRSA, however no additive effect with antibiotic was detected.

The MRSA was treated with PEF (5–20 kV/cm; 100 μs × 8) separately and in combination with antibiotic treatment (1–1000 μg/mL) and the viability was evaluated. The results for inactivation of MRSA using electroporation separately and in combination with vancomycin are presented in Figure 1.

As it can be seen in Figure 1 electroporation can be effectively used for inactivation of *S. aureus* cells, while the threshold for detectable inactivation was observed during 10 kV/cm treatment. As expected, the 20 kV/cm treatment was the most effective due to high energy of the pulses. The strain was resistant to vancomycin and thus, no detectable effect was observed independently on the applied antibiotic concentration. Combination of both treatment methodologies also did not result in additional loss of cell viability. It implies that the inactivation of *S. aureus* cells was induced solely by irreversible electroporation.

**Figure 2:**
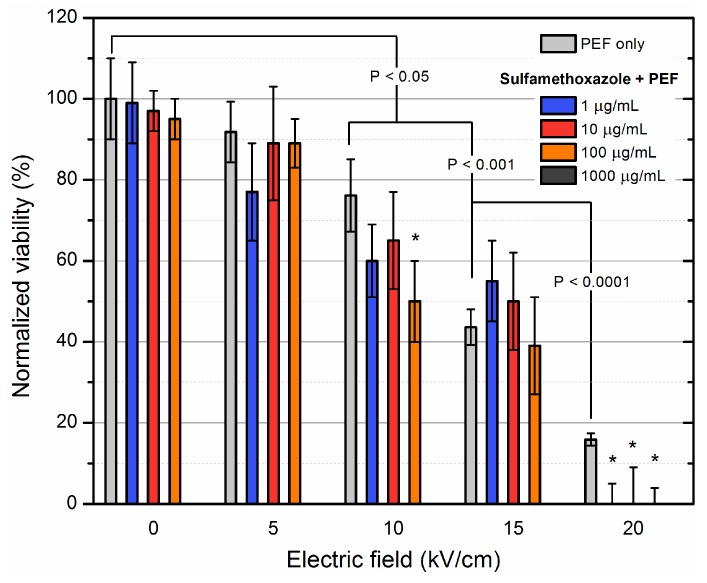
Inactivation of MRSA using electroporation separately and in combination with sulfamethoxazole of varied concentration. Asterisk (*) represents statistically significant (*p* < 0.05) difference versus only pulsed electric field (PEF) treatment. Statistically significant (*p* < 0.05) additive effect with PEF was observed during the 20 kV/cm treatment.

The same protocol was used with sulfamethoxazole and the results are summarized in Figure 2.

As it can be seen in Figure 2 the 1000 μg/mL antibiotic concentration was already sufficient for full eradication of *S. aureus* cells. However, the additive effect was of utmost interest. A statistically significant additive effect was observed during the 10 kV/cm treatment and 100 μg/mL sulfamethoxazole procedure. During 20 kV/cm all of the concentrations of the applied antibiotic resulted in an additive effect with PEF, which implies that PEF can be successfully used for sensitization and full eradication of bacteria.

**Figure 3:**
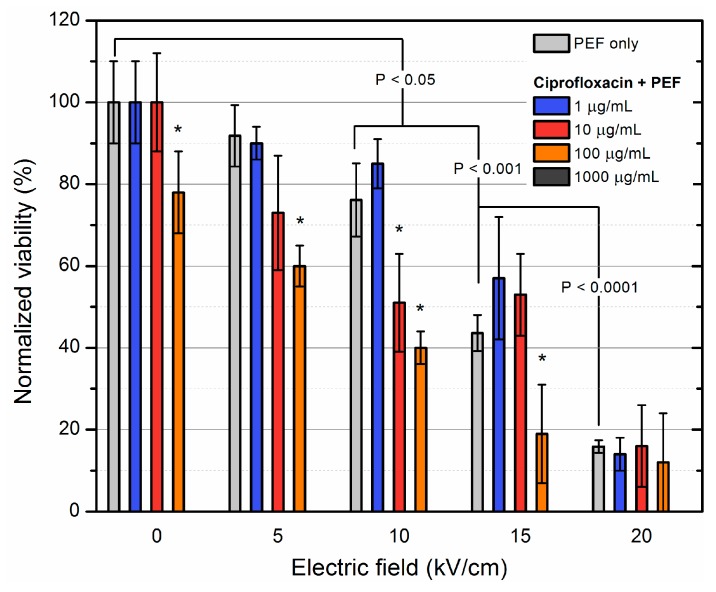
Inactivation of MRSA using electroporation separately and in combination with ciprofloxacin of varied concentration. Asterisk (*) represents statistically significant (*p* < 0.05) difference versus PEF only treatment. Additive effect of PEF with antibiotic was observed at 5 kV/cm already indicating interaction of the antibiotic and permeabilized cell membrane.

The PEF methodology was further investigated with ciprofloxacin. The results are summarized in Figure 3.

Similar tendency was persistent for ciprofloxacin, however the 100 μg/mL concentration was already high enough to result in a detectable inactivation of *S. aureus* even without PEF. At the same time, PEF induced the sensitization to drugs in the 5–15 kV/cm range, which improved the efficacy of the treatment by more than 30%, however saturation at 20 kV/cm was reached. The lowered 5 kV/cm threshold indicates an interaction of the antibiotic with reversibly permeabilized cell membrane.

**Figure 4:**
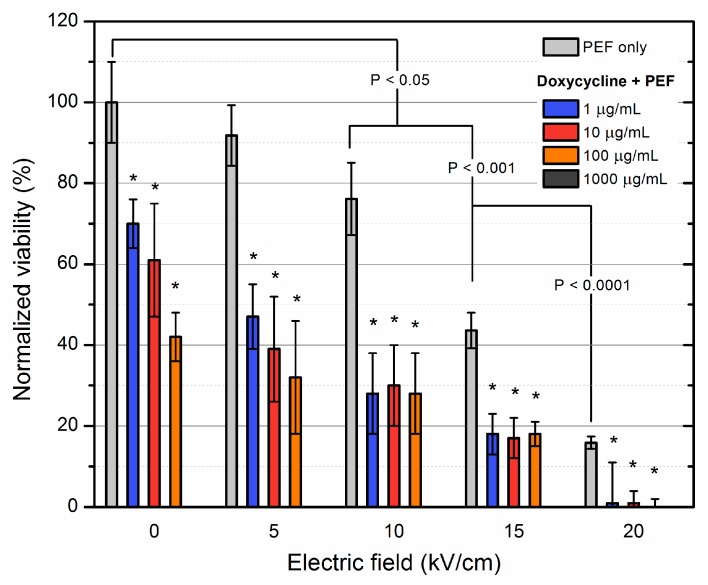
Inactivation of MRSA using electroporation separately and in combination with doxycycline of varied concentration. Asterisk (*) represents statistically significant (*p* < 0.05) difference versus PEF only treatment. The bacteria sensitization phenomenon was detectable in the whole range of applied parameters.

Further, doxycycline was introduced in the study and the results are summarized in Figure 4.

For doxycycline, all of the applied concentrations were effective (resulted in detectable inactivation). Also, the PEF induced sensitization phenomenon was more pronounced. The additive effect was observed in 15, 20 kV/cm PEF for all of the applied concentrations. It can be seen that PEF allows achieving the same inactivation efficacy with up to 1000-fold smaller concentration of antibiotic.

**Figure 5:**
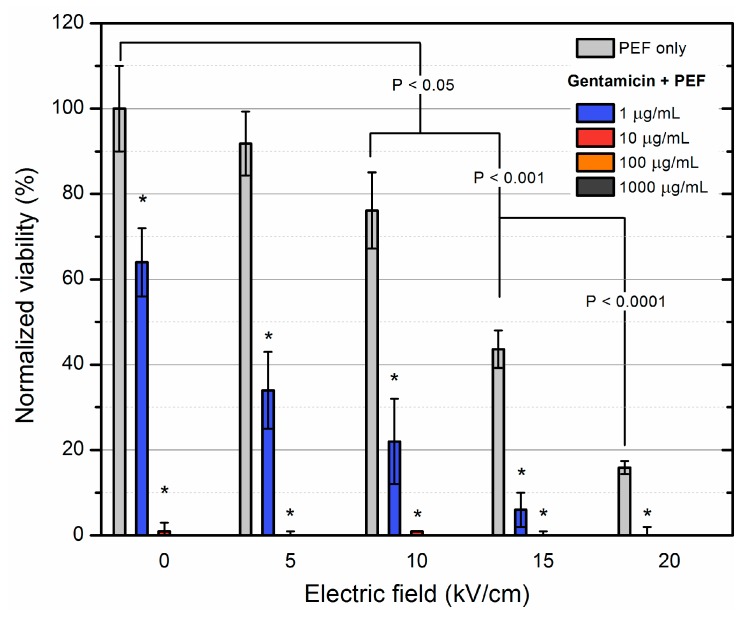
Inactivation of MRSA using electroporation separately and in combination with gentamicin of varied concentration. Asterisk (*) represents statistically significant (*p* < 0.05) difference versus PEF only treatment. Gentamicin was the most effective antibiotic in the study and even the lowest concentration (1 μg/mL) resulted in a significant additive effect for all PEF amplitudes.

Lastly, the additive effect of gentamicin with PEF was evaluated. The results are shown in Figure 5.

MRSA was the most susceptible to gentamicin. All of the concentrations were effective and as a result, the PEF sensitization phenomenon was also detectable in the whole range of the applied amplitudes (5–20 kV/cm). The combination of two methodologies allowed achieving complete inactivation of *S. aureus* cells.

## 3. Discussion

The treatment of bacteria-associated infections is complicated, while antibiotic treatment alone is often inadequate to overcome biofilm infections [8]. Our results show a potential of electroporation to overcome this problem and significantly improve the currently available PEF wound sterilization concept [25] by combination of electroporation with antibiotics and sensitization of MRSA.

The possible mechanism of the observed inactivation by PEF is presumably straightforward and is associated with the physical damage that is caused to the bacterial cells. The integrity and morphology of bacteria are sustained by the cell wall serving as a barrier against the environment, while it is known that PEF induces disorganization with morphological, functional, and mechanical alterations of the cell wall [29]. Additionally, PEF induces reversible (5–10 kV/cm) and non-reversible (15–20 kV/cm) cell membrane permeability increase by creation of pores [18], which also affect passive diffusion of molecules inside the cells. The increase of antimicrobial efficacy is consistent, since the membrane is often a target for improvement of the efficacy of antibiotic treatment [30]. Lastly, the electrophoretic forces during the pulses allow further enhancing of the intracellular diffusion of antibiotics, which is believed to be the main mechanism behind the sensitization phenomenon observed in the low electric field region without electroporation [14]. The high intensity (1000-fold higher compared to [14]) PEF-induced permeabilization of MRSA enables a rapid and effective treatment.

Our results are in agreement with the established electroporation theory [31] and the work of Ravensdale et al., which showed that PEF can be also used to restore the sensitivity of MRSA to antimicrobial peptides [32]. Taking into account that the potency of electroporation for eradication of biofilms was proven recently [26], our presented data indicates that the described bacteria sensitization phenomenon will enable significant improvement of the methodology. According to the Chou and Talalay treatment combination index [33], an additive effect is triggered.

Additionally, we have shown that the sensitization efficacy also depends on the initial susceptibility of the strain to antibiotics. In our case, the MRSA was resistant to vancomycin (VRSA), which eliminates the use of many β-lactams as a treatment option [34]. The mode of action of vancomycin and other glycopeptides involves binding to the D-Ala-D-Ala C-terminal peptide of Lipid II, physically blocking its recognition, and eventual cross-linking, by penicillin-binding proteins [35]. At the same time, the doxycycline (tetracycline class) and gentamicin (aminoglycoside class) were the most effective against MRSA in our study and both showed a definitive additive effect with PEF. Both antibiotics feature a similar mechanism of effect, that is, target protein synthesis inhibition through binding to 30S ribosomal subunit [35,36]. Therefore, the mechanism of resistance is notably related to efflux pumps, while membrane permeabilization (in our case by electroporation) can significantly alter the drug access [37].

In the case of sulfamethoxazole, the MRSA resistance can be influenced by development of the permeability barriers and efflux pumps, thus the additive effect with PEF is in agreement with the currently available knowledge [38]. The quinolone resistance (ciprofloxacin) may be associated with chromosomal mutations that alter the target enzymes or drug accumulation by either decreased uptake or increased efflux, which again, at least partially, allow explanation of the increased sensitivity of MRSA due to the active electrotransfer of drugs inside the cells [39]. Current data do not allow the formation of descriptive conclusions regarding the exact mechanism of the effect and the topic requires further study.

Nevertheless, the defined PEF-induced sensitization phenomenon and inability of microorganisms to form a resistance to PEF are the major advantages, which will be responsible for the interest in the technique. The major disadvantages involve the requirement for the development of electrodes/applicators and high power generators for pulse delivery. Also, electrochemical reactions in the electrode–tissue interface are possible [40]. However, adequate parametrical analysis for induction of sensitization and accurate selection of the antibiotic will allow overcoming or reducing these challenges. Lastly, we believe that nanosecond range electroporation will be more suitable for triggering permeabilization due to better energy control and thus enabling sustaining the effect predominantly in the infected area with little or no ablation of the healthy tissue. The EP method is highly developed in the area of electrochemotherapy where all the same limitations apply and are successfully countered [41]. Since the EP method is considered safe, the acquisition of a fundamental technological and bureaucratic base for in vivo experiments followed by clinical treatment presumably will be faster and easier.

## 4. Materials and Methods

### 4.1. Electroporation Setup

**Figure 6:**
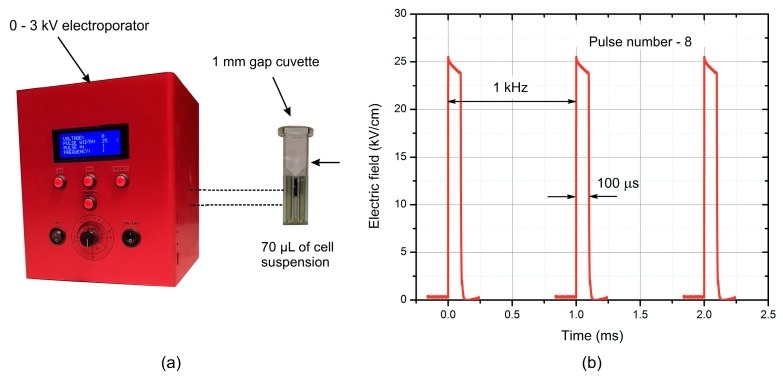
The photo of the setup (**a**) and the waveform of the applied pulses (**b**) that were used for inactivation of *S. aureus*.

The 3 kV, 60 A square wave pulse generator was used for PEF generation (VGTU, Vilnius, Lithuania). A commercially available 1 mm gap electroporation cuvette (VWR International, Radnor, PA, USA) was used as a load. Bursts of 5–20 kV/cm PEF pulses (duration 100 μs × 8) were generated at a pulse repetition frequency of 1 kHz. The photo of the setup and the waveform of the applied pulses are shown in Figure 6.

### 4.2. Bacterial Strain and Electroporation

The methicillin-resistant strain of Staphylococcus aureus (NCRBTL3104) was used in the experiments. It was isolated from the blood of a septic patient in the Laboratory of Microbiology of the Centre of Laboratory Medicine, Vilnius University Hospital Santaros Clinics. The methods were approved by the Lithuanian Bioethics Committee, License Number 24 and informed written consent was obtained from the subject. All applicable international, national and/or institutional ethical guidelines were followed. For isolation of *S. aureus*, the Blood Agar Base (Liofilchem, Roseto degli Abruzzi, Italy) was used. *S. aureus* was identified using MALDI Biotyper 2.3 (Bruker Daltonik GmbH, Bremen, Germany) and stored at −70 °C in Laboratory of Biodeterioration Research of the Nature Research Centre, Vilnius, Lithuania. *S. aureus* was subcultured on nutrient agar (NA) (Liofilchem, Italy). After 48 h growth at 37 °C the bacteria cells suspension was prepared in 1 M sorbitol (ROTH, Karlsruhe, Germany) to a concentration of 10^10^ CFU/mL.

### 4.3. Viability Assay

For the combined (PEF + antibiotic) procedure the suspension of *S. aureus* cells was mixed with different concentrations of antibiotic. Gentamicin sulphate (Sigma-Aldrich, Darmstadt, Germany), doxycycline monohydrate (Alfa Aesar, Karlsruhe, Germany), and vancomycin hidrochloride (Sigma-Aldrich, Germany) were provided by the manufacturers as standard powder and the stock solutions were prepared in sterile water. Sulfamethoxazole (Dr. Ehrenstorfer GmbH, Augsburg, Germany) was provided by the manufacturers as standard powder and the stock solution was prepared in ethanol. Ciprofloxacin (Sigma-Aldrich, Germany) was provided by the manufacturers as standard powder and the stock solution was prepared in 0.1 N HCL. The final concentrations of 1 µg/mL, 10 µg/mL, 100 µg/mL, 1000 µg/mL for gentamicin, doxycycline, ciprofloxacin, sulfamethoxazole and vancomycin were used. The 80 μL cell suspension samples were treated by PEF. After treatment, the samples were incubated at ambient temperature for 24 h. After the 24 h incubation, the *S. aureus* cells were plated on the Mueller-Hinton agar (Liofilchem, Italy). The colony forming units (CFU) were counted after 48 h of incubation at 37 °C. All tests were performed in triplicate.

### 4.4. Statistical Analysis

One-way analysis of variance (ANOVA; *p* < 0.05) was used to compare the viability between the different protocols. Tukey HSD multiple comparison test for the evaluation of the difference was used (*p* < 0.05 was considered statistically significant). The data was post-processed in OriginPro 8.5 software (OriginLab, Northhampton, MA, USA). All experiments have been performed at least in triplicate and the treatment efficacy was expressed as mean ± standard deviation normalized to the untreated control.
